# Unusual Vascular Claudication in a Seronegative Patient: Raynaud's Phenomenon or Atypical Buerger Disease

**DOI:** 10.7759/cureus.116069

**Published:** 2026-09-10

**Authors:** Umar Rehman, Abhinav Shoor, Abdulhaadi Khan, Mufadda Hasan

**Affiliations:** 1 Internal Medicine, Arrowhead Regional Medical Center, Colton, USA; 2 Pulmonary and Critical Care Medicine, Arrowhead Regional Medical Center, Colton, USA

**Keywords:** buerger disease, raynaud phenomenon, seronegative vasculitis, small‑vessel ischemia, thromboangiitis obliterans

## Abstract

We report a diagnostically challenging case of progressive upper‑extremity ischemia in a 31‑year‑old non‑smoking woman whose presentation defied classic criteria for thromboangiitis obliterans (TAO), Raynaud’s phenomenon (RP), or small‑vessel vasculitis. Despite initial imaging suggesting brachial artery stenosis, repeat computed tomography angiography (CTA), ultrasound, and angiography demonstrated patent proximal vessels with sluggish distal forearm flow and diminutive radial and ulnar arteries at the wrist, consistent with terminal small‑vessel disease. Extensive hypercoagulable and vasculitis workup was unrevealing, and inflammatory markers remained normal. The patient’s symptoms progressed from intermittent discoloration and pain to persistent ischemia, ultimately improving only after prostacyclin infusion, suggesting underlying microvascular dysfunction. Her atypical demographic profile-female sex, absence of tobacco exposure, isolated upper‑extremity involvement-and lack of systemic features created diagnostic ambiguity, raising consideration of atypical TAO, severe secondary RP, or seronegative vasculitis. This case highlights the importance of maintaining broad diagnostic suspicion and individualized management in patients with unexplained distal ischemia and nondiagnostic serologic and imaging findings.

## Introduction

Buerger disease, or thromboangiitis obliterans (TAO), is a progressive, non‑atherosclerotic, segmental inflammatory disorder that primarily involves the small and medium‑sized arteries of the upper and lower extremities [1]. It occurs more frequently in men than women (approximately 3:1) and is most common in individuals aged 20-45 years [2]. Unlike atherosclerotic disease, TAO involves all three layers of the arterial wall. Early symptoms often include claudication of the feet, legs, hands, or arms, which can initially be mistaken for musculoskeletal or neuromuscular conditions [2]. As the disease advances, patients may develop calf claudication followed by ischemic rest pain and ulcerations affecting the toes, feet, or fingers. Vasospastic episodes resembling Raynaud's phenomenon (RP) may also occur [1,2].

Proposed mechanisms include immunologic dysfunction and hypersensitivity to tobacco components, leading to increased cellular reactivity to type‑1 and type‑3 collagen, impaired endothelium‑dependent vasodilation, and elevated anti‑endothelial cell antibody levels [2]. These processes contribute to vessel wall hypercellularity, recurrent luminal narrowing, and organized thrombus formation with subsequent fibrosis [2]. Although the exact cause of TAO remains unclear, tobacco exposure is essential for both the initiation and progression of the disease [2]. In this case, we describe an unusual case of vascular claudication of unclear etiology, potentially representing an atypical Buerger‑like disease associated with RP or a seronegative vasculitis with asymmetric involvement.

## Case presentation

A 31‑year‑old woman with a history of endometriosis and prior left lower‑extremity deep vein thrombosis and pulmonary embolism (right upper lobe), previously treated with apixaban, presented with one week of progressively worsening right upper‑extremity pain, numbness, and weakness (Figure 1). Repeat bilateral venous Doppler ultrasound prior to presentation showed no evidence of residual deep vein thrombosis.

**Figure 1:**
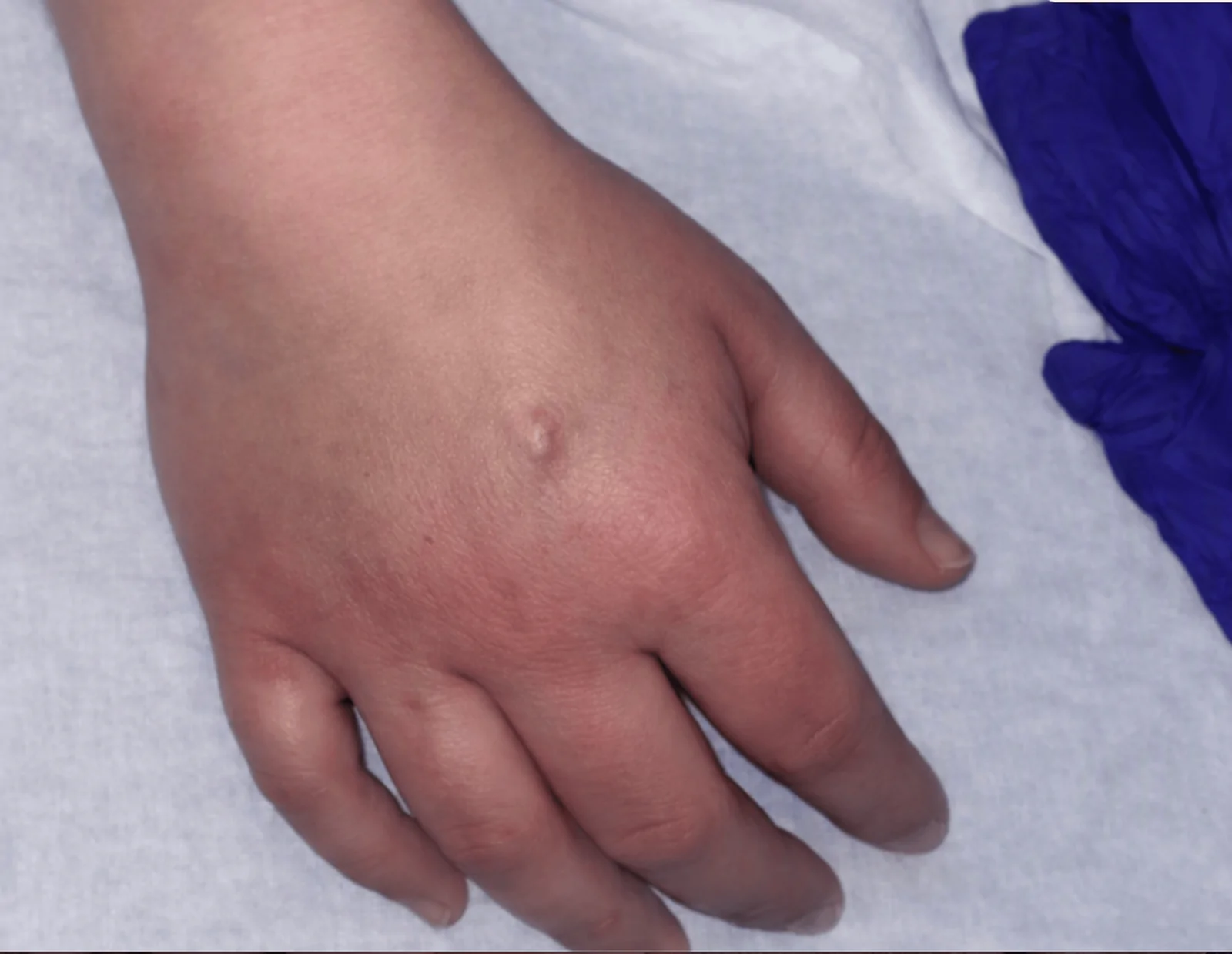
Photograph of Right Hand Prior to Computed Tomography Right upper extremity (RUE) of the patient presenting with one week of ascending right‑arm pain, numbness, and weakness. The image demonstrates purplish discoloration and swelling of the right hand, consistent with the patient’s initial symptoms of sharp, intermittent pain, tingling, and episodic cyanosis. She was evaluated at an outside hospital at that time for these symptoms, which progressively worsened over the following week.

She initially sought care at an outside hospital for sharp, intermittent pain, tingling, and purplish discoloration of the right hand. Computed tomography angiography (CTA) performed there reportedly demonstrated hemodynamically significant stenosis of the right brachial artery, and she was started on heparin. Upon transfer to our institution for vascular surgery evaluation, repeat CTA showed no arterial stenosis or occlusion, and she was discharged on aspirin 81 mg with outpatient follow‑up.

Three days later, she re‑presented to another hospital with worsening pain, weakness, and numbness of the right upper extremity. The discoloration, initially intermittent and limited to the hand, had become constant and ascended to the mid‑forearm. She also reported transient blurry vision in the right eye at symptom onset, which had resolved. She denied systemic symptoms including fever, chills, chest pain, dyspnea, abdominal pain, rash, oral ulcers, or joint pain. She had no smoking history. A prior urine toxicology screen had been positive for barbiturates, fentanyl, and benzodiazepines, all of which may have been administered during prior medical care; there was no evidence of active substance use contributing to her presentation. CTA and arterial ultrasound again demonstrated patent arteries with normal flow, and venous ultrasound showed no thrombosis.

Vascular surgery performed a right upper‑extremity angiogram, which demonstrated patent subclavian, axillary, brachial, radial, ulnar, and interosseous arteries without focal stenosis or occlusion. However, sluggish flow was observed throughout the forearm, and both the radial and ulnar arteries became diminutive at the wrist, with scant filling of the hand, consistent with distal microvascular dysfunction or ischemia (Figures 2A, 2B). These findings were non‑specific and did not definitively indicate thromboangiitis obliterans, RP, or vasculitis.

**Figure 2:**
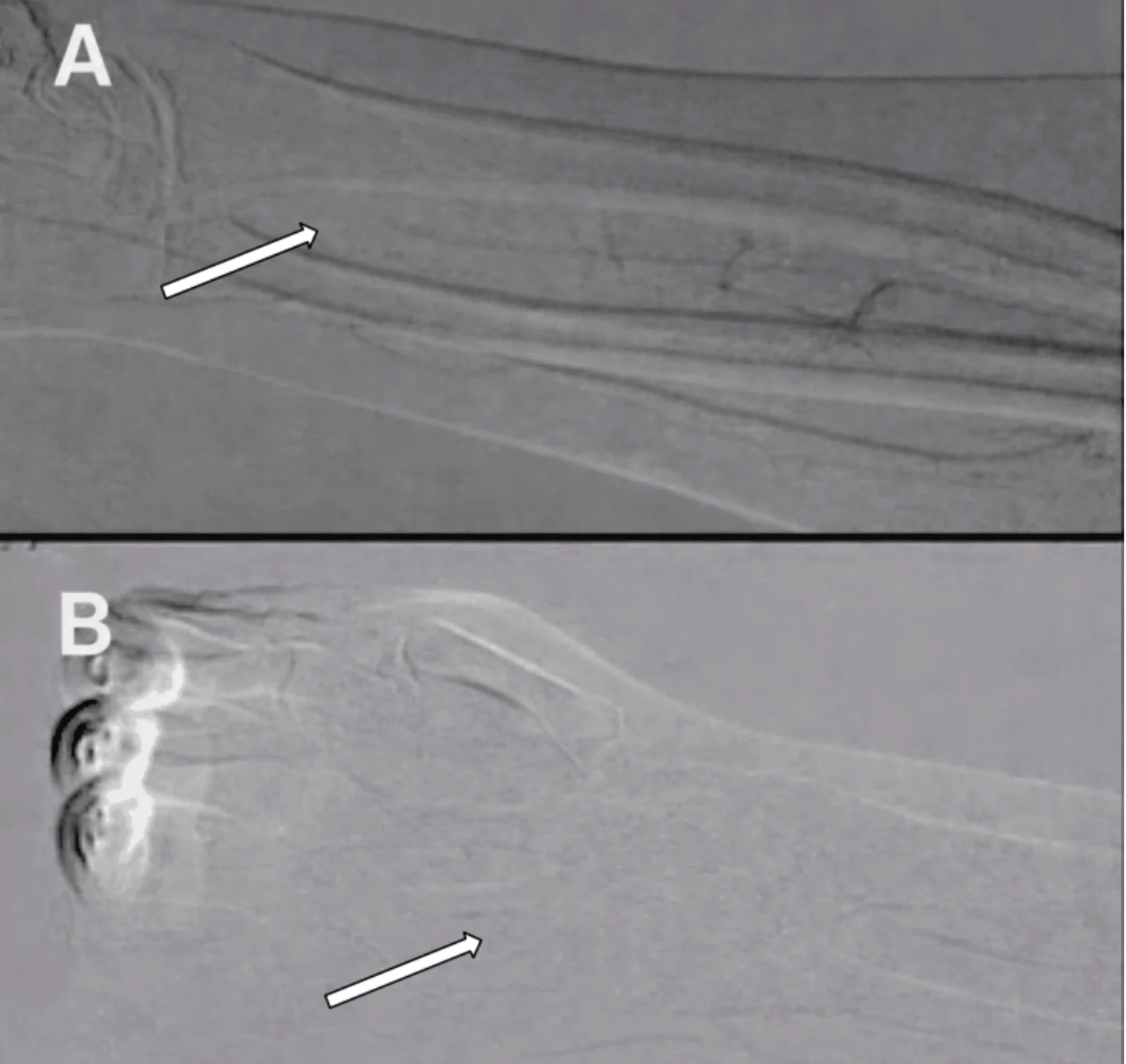
Right Upper Extermity Angiogram: Forearm, Wrist, and Hand (A) Sluggish flow is noted throughout the forearm (white arrow in 2A), with tapering of the distal vessels consistent with impaired runoff. (B) Scant filling of the radial arteries is observed (white arrow in 2B), demonstrating diminutive flow at the level of the wrist, suggestive of distal small‑vessel disease.

Rheumatology was consulted for suspected small‑vessel disease. A comprehensive evaluation was performed. The hypercoagulable workup was negative, including antiphospholipid antibodies, factor V Leiden mutation, and factor II (prothrombin) mutation. The vasculitis evaluation was unrevealing, with normal erythrocyte sedimentation rate (ESR) and C-reactive protein (CRP), no clinical evidence of mononeuritis multiplex, no palpable purpura, no inflammatory arthritis, and no pulmonary involvement.

Given concern for seronegative vasculitis, she was started on intravenous corticosteroids, after which she reported mild improvement over two days. Per rheumatology recommendations, epoprostenol (Flolan) infusion was initiated at 2 ng/kg/min, and she was transferred to the intensive care unit for close monitoring due to the risk of hypotension associated with prostacyclin therapy. Over four days of infusion, she experienced significant improvement in pain, discoloration, and swelling (Figure 3). She remained stable on the medical ward thereafter, with continued reduction in forearm edema and improved perfusion.

**Figure 3:**
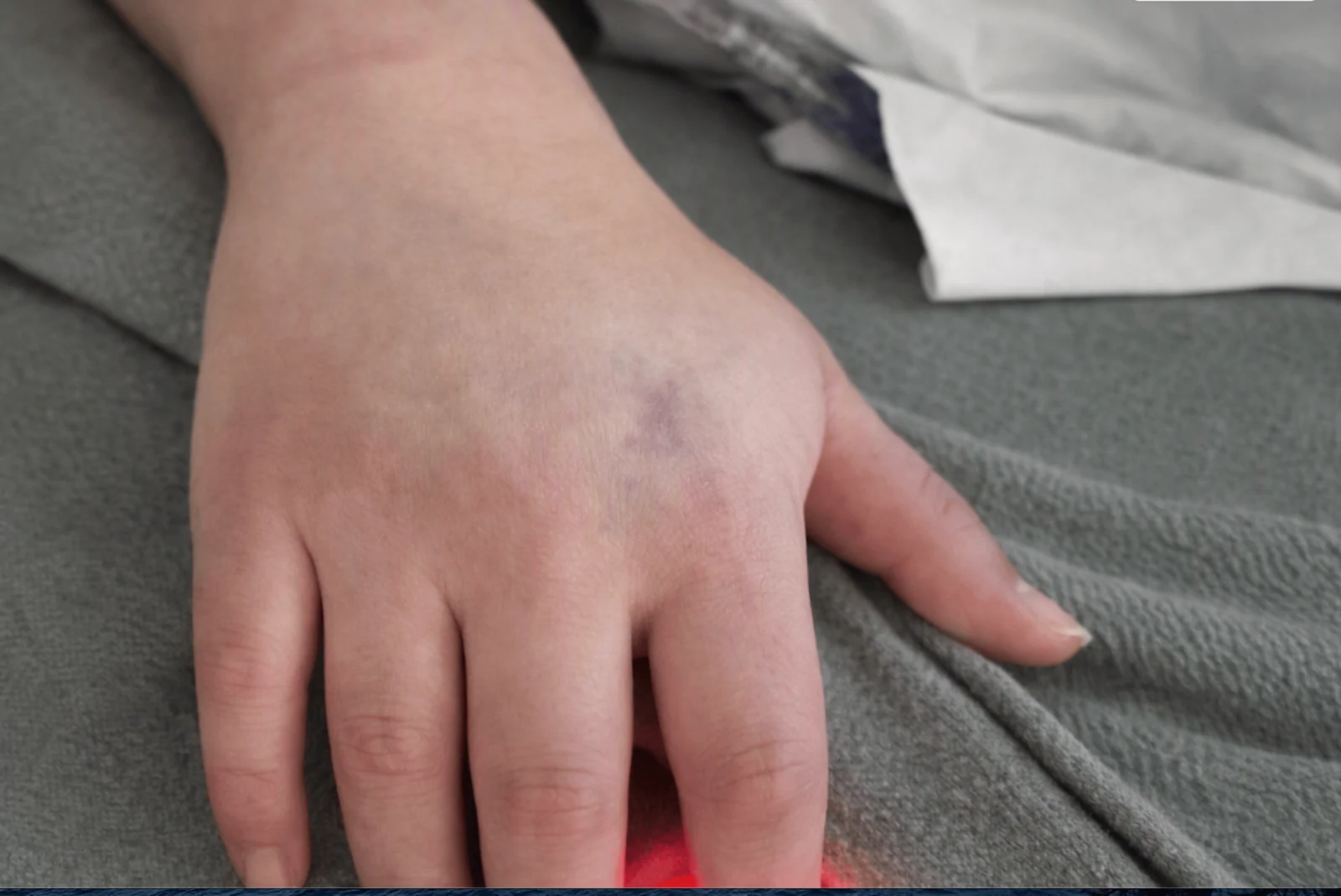
Photograph of Right Hand Post-Treatment 2 days of Intravenous (IV) Steroids and 2 days of IV Flolan This photograph shows the patient’s right hand after two days of IV steroids followed by two days of IV Flolan therapy. There is a clear reduction in erythema, swelling, and, most notably, the purplish discoloration that was prominent at the time of admission.

She was started on cilostazol 25 mg twice daily, with instructions to use topical nitroglycerin paste if ischemic symptoms recurred. Follow‑up after discharge was arranged to monitor for recurrence of vasospastic or ischemic symptoms and to reassess the need for ongoing vasodilator therapy.

## Discussion

This case concerns unexplained distal upper‑extremity ischemia in a young woman without tobacco exposure and without serologic or clinical evidence of systemic vasculitis. From the outset, the diagnostic uncertainty spans several potential etiologies-including TAO, secondary RP, and small‑vessel vasculitis-none of which fully account for the totality of her presentation.

Classic TAO is a segmental, non‑atherosclerotic occlusive disease of small‑ and medium‑sized vessels, historically described in young male smokers [2]. Although tobacco exposure is strongly associated with TAO and considered central to its pathogenesis, rare cases in nonsmokers have been reported, suggesting that tobacco use, while highly characteristic, may not be essential for disease development. Typical manifestations include distal extremity ischemia, migratory thrombophlebitis, ischemic ulcers, and gangrene, and diagnostic criteria emphasize exclusion of autoimmune, embolic, or connective‑tissue diseases [2].

Our patient shares several TAO‑like features: young age, distal ischemia with preserved proximal arterial flow on angiography (Figures 2A, 2B), and negative autoimmune serologies. The absence of inflammatory markers and autoantibodies is particularly helpful when distinguishing TAO from other arteritides [2]. However, multiple aspects of her presentation diverge from classic TAO: she is female, a lifelong non‑smoker, and exhibits isolated upper‑extremity involvement, whereas TAO more commonly affects the lower extremities [2]. Additionally, angiography demonstrated sluggish forearm flow rather than the classic segmental occlusions typically associated with TAO (Figures 2A, 2B) [2]. These atypical features prevent confident classification as TAO.

Secondary RP represents another potential explanation. RP is a vasospastic disorder triggered by cold exposure or emotional stress, producing triphasic color changes and sometimes progressing to digital ischemia, ulceration, or gangrene [1]. Secondary RP may arise from numerous systemic conditions, including TAO, and severe cases may respond to prostacyclin therapy [1]. Our patient exhibited RP‑like symptoms, including discoloration, pain, numbness, and a positive response to prostacyclin (Figure 3). However, her symptoms evolved from intermittent vasospasm to persistent ischemia without a clear cold‑triggered pattern, making RP alone insufficient to explain the full clinical picture.

Small‑vessel vasculitis was also considered. Vasculitis causes inflammatory destruction of vessel walls, leading to narrowing, occlusion, and tissue ischemia, and small‑vessel vasculitides typically present with multisystem involvement-purpura, glomerulonephritis, pulmonary disease, neuropathy-and elevated inflammatory markers or positive serologies [3,4]. Our patient lacked systemic manifestations, had normal inflammatory markers, and demonstrated no serologic evidence of vasculitis, making this diagnosis less likely.

The pathophysiology of TAO centers on endothelial injury, platelet activation, leukocyte recruitment, inflammation, and thrombus formation. Histologically, TAO is characterized by an intact internal elastic lamina and a highly inflammatory thrombus composed of neutrophils, microabscesses, and giant cells [2]. Immune dysregulation-including anti‑endothelial cell antibodies and T‑cell activation-has also been implicated, although autoantibodies are typically absent and ESR/CRP are often normal [2]. Impaired vasodilation may explain our patient’s marked improvement with prostacyclin, suggesting microvascular dysfunction consistent with TAO physiology despite the absence of tobacco exposure.

In contrast, RP pathophysiology involves an imbalance favoring vasoconstrictors (e.g., endothelin‑1, catecholamines) over vasodilators (e.g., nitric oxide, prostacyclin). Recurrent vasospasm in secondary RP can lead to structural vascular remodeling, intimal proliferation, luminal narrowing, and chronic ischemia-features that can mimic small‑vessel occlusive diseases such as TAO [1]. Neurovascular dysregulation may further contribute to hyperreactive vessels. The patient’s response to prostacyclin may reflect correction of this vasoconstrictor‑vasodilator imbalance.

Several similar cases have been described. Shima et al. reported a non‑smoking woman with severe limb ischemia initially suspected to have polyarteritis nodosa or antiphospholipid syndrome; she failed immunosuppressive and anticoagulant therapy, and post‑amputation pathology confirmed TAO [3,5,6]. Varim & Tatli described upper‑extremity TAO requiring revascularization in a male smoker [5], while Khan et al. reported upper‑extremity digital ischemia due to TAO in a pregnant non‑smoker [3]. These cases highlight that although uncommon, TAO can occur in nonsmokers and in the upper extremities, supporting its inclusion in the differential.

Ultimately, this case does not conform neatly to TAO, RP, or vasculitis. Instead, it demonstrates overlapping features without meeting full diagnostic criteria for any single entity. The combination of female sex, absence of tobacco exposure, negative serologic evaluation, lack of systemic vasculitis features, isolated upper‑extremity involvement, and significant improvement with prostacyclin therapy creates persistent diagnostic ambiguity. Without tissue biopsy, a definitive diagnosis remains elusive, underscoring the need for heightened clinical suspicion and individualized management in atypical presentations of small‑vessel ischemia.

## Conclusions

This case underscores the complexity of diagnosing small‑vessel ischemia in patients who do not fit classic disease patterns. Despite sharing select features with thromboangiitis obliterans, RP, and small‑vessel vasculitis, the patient lacked the defining criteria for any single entity, resulting in a broad and challenging differential. Her absence of tobacco exposure, normal serologic evaluation, isolated upper‑extremity involvement, and robust response to prostacyclin highlight the possibility of an atypical, microvascular‑driven process that mimics elements of each condition without fully conforming to established diagnostic frameworks. This presentation reinforces the importance of maintaining diagnostic flexibility, integrating angiographic findings with clinical evolution, and considering individualized therapeutic trials-particularly vasodilatory agents-in unexplained distal ischemia. Ultimately, atypical cases such as this emphasize the need for heightened clinical suspicion and may prompt further investigation into non‑traditional or seronegative variants of occlusive small‑vessel disease.
